# The impact of PD-L1 polymorphisms on the efficacy of immune checkpoint inhibitors depends on the tumor proportion score: a retrospective study

**DOI:** 10.1007/s00432-024-06081-x

**Published:** 2025-02-04

**Authors:** Keiichiro Suminaga, Takashi Nomizo, Hironori Yoshida, Hiroaki Ozasa

**Affiliations:** 1 Department of Respiratory Medicine, Graduate School of Medicine, Kyoto University, 54 Kawaharacho, Shogoin, Sakyo-Ku, Kyoto, 606-8507 Japan; 2 Department of Therapeutic Oncology, Graduate School of Medicine, Kyoto University, 54 Kawaharacho, Shogoin, Sakyo-Ku, Kyoto, 606-8507 Japan

**Keywords:** Programmed death-ligand 1, Single nucleotide polymorphism, Progression free survival, Immune checkpoint inhibitor

## Abstract

**Purpose:**

This study aims to clarify the relationship between rs2282055, a single-nucleotide polymorphism (SNP) in programmed death-ligand 1 (PD-L1), and TPS. Polcaro et al. (2024) showed that rs822336, a SNP in PD-L1, predicts the effect of immune checkpoint inhibitors (ICIs). However, the study did not show a relationship between rs822336 and the tumor proportion score (TPS), which is currently used as a primary marker. Therefore, we examined this relationship.

**Method:**

Patients treated with immune checkpoint inhibitor monotherapy for non-small cell lung cancer at Kyoto University Hospital until January 2023, with TPS data and biological specimens available for SNP measurement, were eligible for this study. Genomic DNA was extracted from peripheral blood leukocytes. We used rs2282055, which is in linkage disequilibrium with rs822336, instead of rs822336, because of its distribution in the Asian patient population. We retrospectively extracted data on age, sex, smoking history, driver mutations, TPS, progression-free survival (PFS), and best response to ICI from medical records.

**Result:**

The rs2282055 T/T genotype was associated with significantly better PFS in the TPS-negative population than in the other genotypes. In contrast, no differences were observed in TPS-positive patients.

**Conclusion:**

The rs2282055 genotype may help in selecting cases from the TPS-negative patient population that may benefit from ICI therapy.

## Introduction

Immune checkpoint inhibitors (ICIs) have greatly improved the prognosis of lung cancer patients compared to conventional anticancer drugs (Borghaei et al. 2015; Fehrenbacher 2016; Herbst et al. 2016). On the other hand, the best markers for predicting the effects of ICI are not clear. The tumor proportion score (TPS) is now widely used as a marker to predict the effect of ICIs. It has been reported that ICI is more effective when TPS is highly expressed (Hara et al. 2021). However, several problems have been reported with TPS, which has been reported to vary by metastatic site, even for the same tumor. It has also been noted that TPS changes over time (Mansfield et al. 2016; Schoenfeld et al. 2020). These problems reduce the credibility of TPS as a biomarker. Previous clinical trials have failed to establish the efficacy of ICI over conventional chemotherapy in TPS-negative populations, so ICI is not recommended as initial therapy in these patient groups (Borghaei et al. 2015; Garon et al. 2015). However, there are cases among these patient groups that respond to ICI, and methods are needed in clinical practice to avoid missing such patients.

Single nucleotide polymorphisms (SNPs) have recently received attention as markers. There were no spatial or temporal variations in the SNPs, which seemed to eliminate the shortcomings observed in the TPS. Polcaro et al. reported that rs822336, a SNP in programmed death-ligand 1 (PD-L1), predicts response to treatment with ICI (Polcaro et al. 2024). The authors proposed rs822336 as an alternative time-invariant marker for TPS, considering its explicit mechanism of action. They showed that in the presence of allele C in rs822336, PD-L1 transcription is regulated by the binding of both C/EBPβ and NFIC, which leads to upregulation of PD-L1 expression in the presence of interferon gamma. Nevertheless, its relationship to TPS, the most frequently used marker today, was unclear. Therefore, we first planned to examine rs822336, including its relationship with TPS. However, allele frequencies are known to vary by race, and the low frequency of rs822336 allele C in Asians made rs822336 unsuitable for consideration in Asians. We therefore examined rs2282055, which is in linkage disequilibrium with rs822336, as an alternative.

## Method

Patients diagnosed histologically or cytologically with non-small cell lung cancer and treated with ICI monotherapy at Kyoto University Hospital, between December 2015 and January 2023, were eligible. Patients without TPS measurements during their clinical course or lacking preserved peripheral blood samples for SNP analysis were excluded. The initial dose of ICIs was 3 mg/kg every two weeks for nivolumab, 1200 mg/body every three weeks for atezolizumab, and 200 mg/body every three weeks or 400 mg/body every six weeks for pembrolizumab, respectively. We defined PFS as the time from the initiation of ICIs administration to the date of disease progression, or death. The disease progression was evaluated using computed tomography, according to the Response Evaluation Criteria for Solid Tumors (RECIST). We investigated the relationship between PD-L1 SNPs and PFS of ICI. The collected data included age, sex, smoking history, histology, driver mutation, and TPS. The survival curve was estimated using the Kaplan–Meier method and compared using the log-rank test. Statistical significance was set at *p* < 0.05. All statistical analyses and data processing were performed using the JMP 17 (SAS Institute Inc., Cary, NC, USA) and GraphPad Prism 10 (GraphPad Software, CA, USA) software.

We collected a 10-mL peripheral blood sample from each patient. SNP genotyping was performed as described in our previous study in detail (Nomizo et al. 2017). Briefly, genomic DNA was extracted from peripheral blood samples using Gene Prep Star NA-480 (Kurabo, Osaka, Japan). Genotyping was performed using the TaqMan genotyping assay (Applied Biosystems, Foster City, CA, USA) and TaqMan genotyping master mix (Applied Biosystems), and analyzed using an Applied Biosystems 7300 Real-Time polymerase chain reaction System (Applied Biosystems). The polymerase chain reaction (PCR) solution consisted of 0.8 µl of DNA sample, 10 µl of TaqMan genotyping master mix, 0.4 µl of TaqMan genotyping assay primer probe mix, and 8.8 µl of nuclease-free water. The baseline fluorescence measurements were taken at 25 °C, followed by the following PCR protocol: incubation of samples at 95 °C for 10 min, 40 cycles of denaturation at 92 °C for 15 s, and annealing and extension at 60 °C for 1 min, with a final fluorescence measurement at 60 °C. The TaqMan genotyping assay primer probe mix for the PD-L1 SNP rs822336 and rs2282055 were purchased from Applied Biosystems.

## Results

**Fig. 1:**
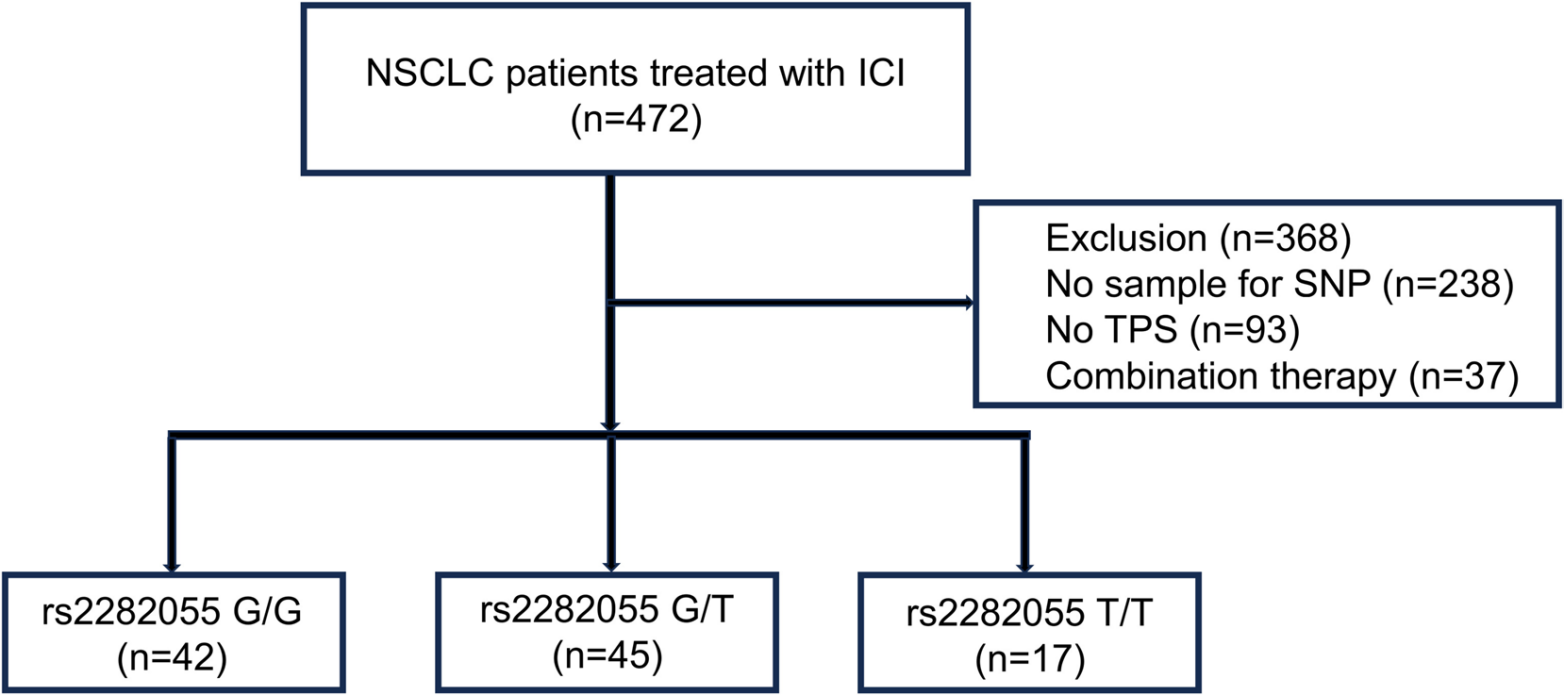
Patient disposition

One hundred and four patients were included in the study (Fig. 1). The patient characteristics are summarized in Table 1. No differences in patient background by SNP were observed. We first examined rs822336 as Polcaro’s previous paper (Polcaro et al. 2024), but because of the low frequency of the rs822336 allele C in the Japanese population, we additionally examined rs2282055, which is in linkage disequilibrium with rs822336. There were 56, 42, and 6 patients with rs822336 G/G, G/C, and C/C, respectively. On the other hand, there were 42, 45, and 17 patients with rs2282055 G/G, G/T, and T/T, respectively. Sixty-four percent of the eligible patients had adenocarcinoma and 21% had driver mutations. The number of patients was 46, 36, 22 for TPS < 1%, 1–49%, ≥ 50%, respectively. The number of patients was 70, 24, 10 for nivolumab, pembrolizumab, atezolizumab, respectively. Treatment lines ranged from first-line to tenth-line therapy, but 70 patients received ICI before third-line therapy.

**Table 1:** Characteristics of patients

	rs2282055 (*n* = 104)		
	G/G or G/T (*n* = 87)	T/T (*n* = 17)	*p* value
Median age (range)	71 (42–84)	69 (54–84)	0.79
Sex			
Male	58 (66.7%)	10 (58.8%)	0.58
Female	29 (33.3%)	7 (41.2%)	
Smoking status			
Never	25 (28.7%)	5 (29.4%)	0.91
Currently/formerly	62 (71.3%)	12 (70.6%)	
Histology			
Adenocarcinoma	54 (62.1%)	13 (76.5%)	0.42
Squamous	23 (26.4%)	2 (11.8%)	
Others	10 (11.5%)	2 (11.8%)	
Treatment line			
1	27 (31.0%)	5 (29.4%)	0.91
2	31 (35.6%)	7 (41.2%)	
≥ 3	29 (33.3%)	5 (29.4%)	

**Fig. 2:**
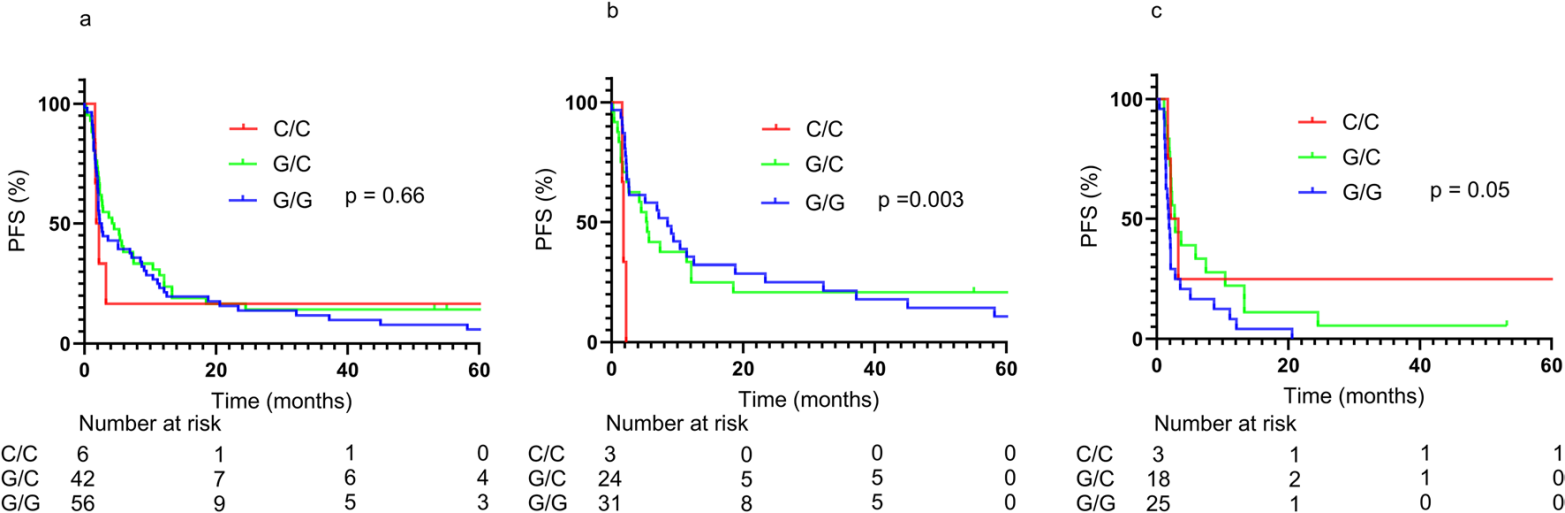
The effect of rs822336 as a PFS marker varied with TPS. Whole group (**a**), TPS positive group (**b**), and TPS negative group (**c**)

**Fig. 3:**
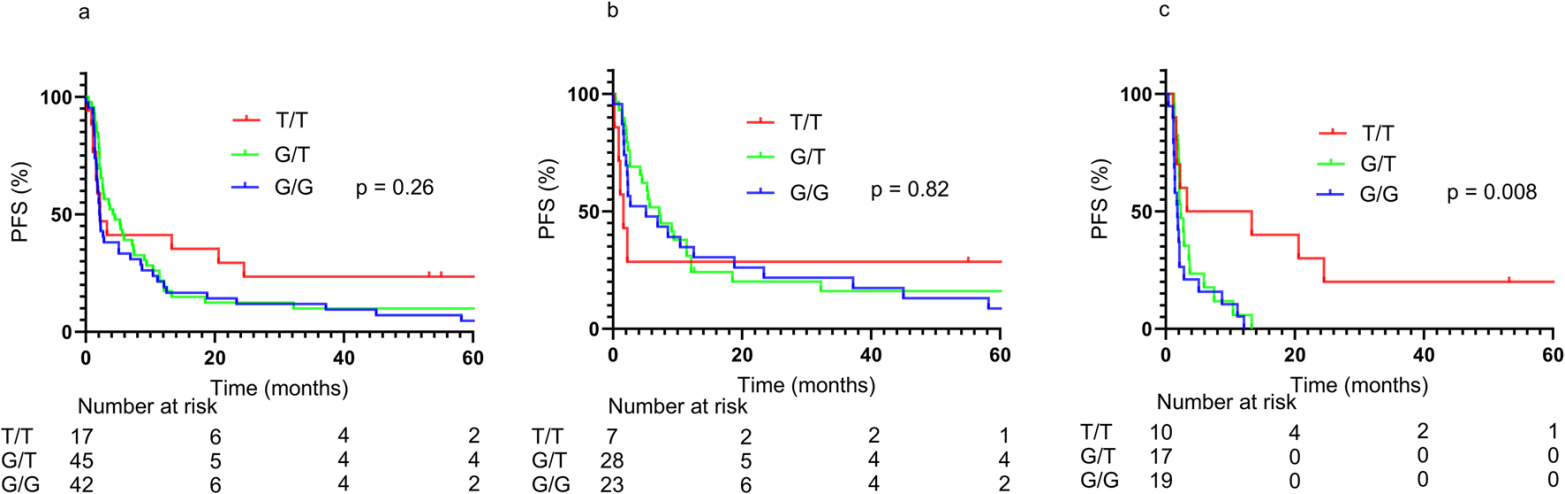
The effect of rs2282055 as a PFS marker varied with TPS. Whole group (**a**), TPS positive group (**b**), TPS negative group (**c**)

**Fig. 4:**
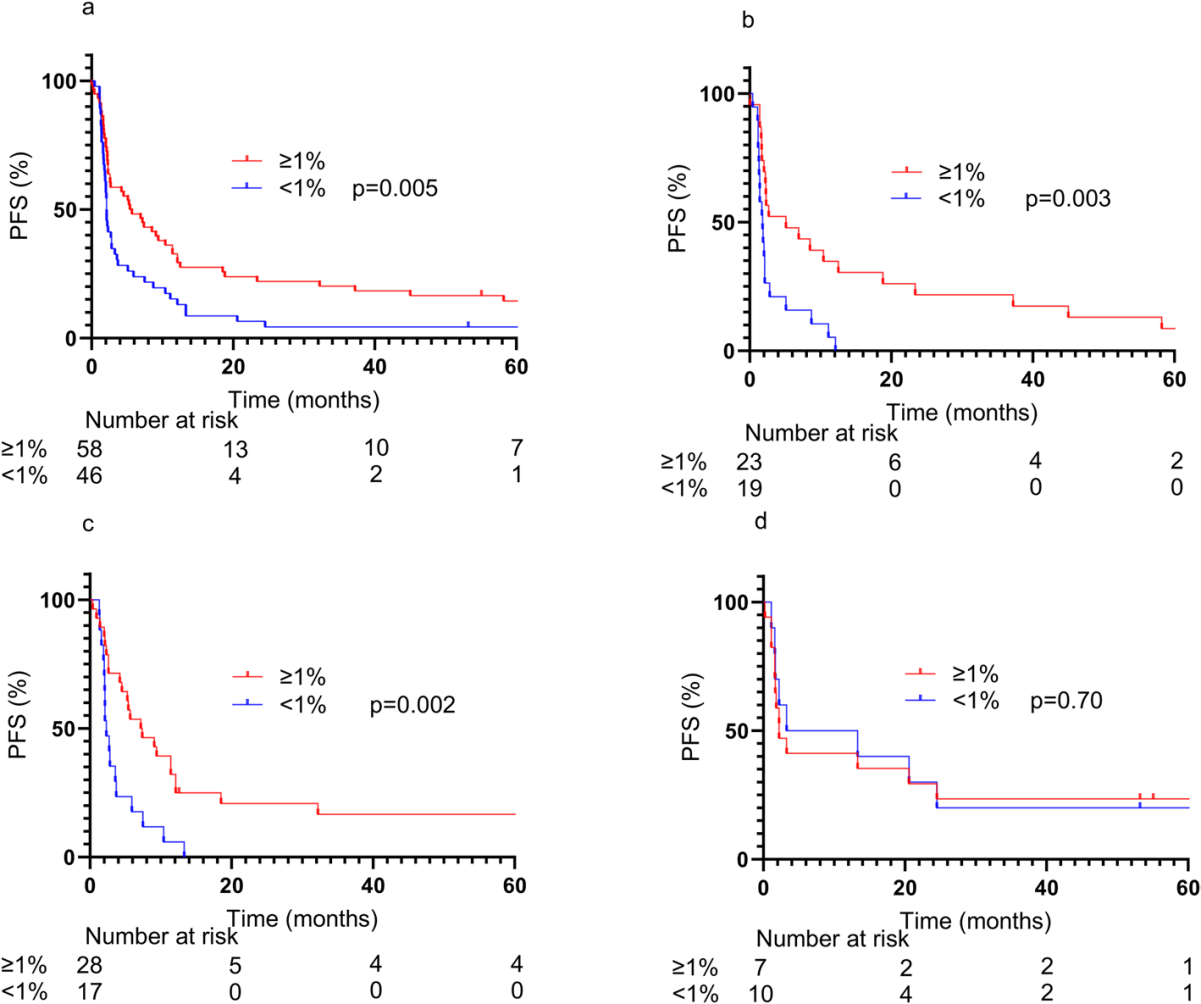
The effectiveness of TPS as a marker depends on genotype. Whole group (**a**), rs2282055 G/G (**b**), rs2282055 G/T (**c**), rs2282055 T/T (**d**)

Unlike previous reports, rs822336 was not associated with PFS in the overall population (Fig. 2a). On the other hand, in the TPS-negative population, a trend toward longer PFS was observed in the genotype C/C population, as previously reported (Fig. 2c). A similar trend was observed with rs2282055, which was added to solve the problem of low frequency of rs822336 allele C. In the overall population, PFS tended to be better in genotype T/T, although not statistically significant (Fig. 3a). In the TPS-negative population, genotype T/T showed clearly better PFS than other genotypes (Fig. 3c). The rs2282055 allele T was in linkage disequilibrium with the rs822336 allele C, and this result was consistent with that. No correlation was found between the rs2282055 genotype and TPS (Table 2). In the overall population, TPS-positive patients tended to have better PFS than TPS-negative patients, but in patients with rs2282055 T/T, TPS-negative patients had PFS comparable to the positive patients (Fig. 4d).

**Table 2:** Relationship between SNP and TPS

TPS	rs2282055			*p* value
	G/G (*n* = 42)	G/T (*n* = 45)	T/T (*n* = 17)	
< 1%	19 (45.2%)	17 (37.8%)	10 (58.8%)	0.64
1–49%	15 (35.7%)	16 (35.6%)	5 (29.4%)	
≥ 50%	8 (19.0%)	12 (26.7%)	2 (11.8%)	

*TPS* Tumor proportion score

## Discussion

This study showed that PD-L1 SNP rs2282055 correlates with PFS specifically in a TPS-negative population. We also showed that rs2282055 is a marker that is independent of TPS. Patients with rs2282055 T/T had the same PFS as those with positive TPS even though they were TPS negative. The results show that combining rs2282055 with TPS can unearth a wider range of patients who will respond to ICI.

It has been reported that rs2282055 is associated with the frequency of immune related adverse events in head and neck cancer, and with the risk and prognosis of colorectal cancer (Tanaka et al. 2023; Cevik et al. 2022). In those reports, it was speculated that an association with PD-L1 expression may have caused the result, but the mechanism was not clarified. Polcaro et al. reported a mechanism for the association between rs822336 and elevated PD-L1 expression, and further noted that ICI was more effective in the rs822336 C/C patient group (Polcaro et al. 2024). The rs2282055 is in linkage disequilibrium with rs822336, and the mechanism by which rs2282055 is associated with the effects of ICI is presumably similar to that shown by Polcaro et al.

It is widely known that patients with high TPS are more likely to respond to ICI, and TPS is the only marker currently used in clinical practice (Topalian et al. 2012; Aguilar et al. 2019). However, heterogeneity has been noted in PD-L1 expression, making it difficult to predict the full ICI effect by itself (Munari et al. 2018); and various markers have been studied as replacements for TPS (Wu et al. 2021; Duchemann et al. 2020). By using this study, we believe that rs2282055 can be used to pick up a patient population that responds to ICI from the previously underestimated TPS-negative population. We believe that this could have major clinical implications.

Our study has some limitations. Polcaro et al. showed that rs822336, a SNP in PD-L1, is a predictive marker of ICI efficacy, along with its mechanism (Polcaro et al. 2024). The results of the present study differs from those shown by Polcaro et al. This is probably due to the fact that the frequency of rs822336 allele C is lower in Asians than in Europeans. The rs822336 and rs2282055 are in linkage disequilibrium and show similar trends in this study. This study does not examine the mechanism by which rs2282055 reflects the effect of ICI. We think that this is due to the fact that it is in linkage disequilibrium with rs822336, for which Polcaro et al. showed a mechanism. However, in consideration of actual clinical use, it is necessary to select which SNPs to use, taking into account differences in allele frequencies by race. Our study is a single-center, retrospective study. It is hoped that it will be followed by large, multicenter, multinational studies in the future.

## Data Availability

No datasets were generated or analysed during the current study.

## Abbreviations

SNP: Single nucleotide polymorphism
PD-L1: Programmed death ligand 1
ICI: Immune checkpoint inhibitor
TPS: Tumor proportion score
PFS: Progression free survival
